# Uncovering heterogeneous cognitive trajectories in mild cognitive impairment: a data-driven approach

**DOI:** 10.1186/s13195-023-01205-w

**Published:** 2023-03-20

**Authors:** Xiwu Wang, Teng Ye, Wenjun Zhou, Jie Zhang, for the Alzheimer’s Disease Neuroimaging Initiative

**Affiliations:** 1 Department of Psychiatry, Wenzhou Seventh People’s Hospital, Wenzhou, China; 2 Department of Ultrasound, The First Affiliated Hospital of Wenzhou Medical University, Wenzhou, China; 3 Research and Development, Hangzhou Shansier Medical Technologies Co., Ltd., Hangzhou, China; 4 Department of Data Science, Hangzhou Shansier Medical Technologies Co., Ltd., Hangzhou, China

**Keywords:** Mild cognitive impairment, Clustering, Heterogeneity, Cognitive trajectory, Longitudinal study

## Abstract

**Background:**

Given the complex and progressive nature of mild cognitive impairment (MCI), the ability to delineate and understand the heterogeneous cognitive trajectories is crucial for developing personalized medicine and informing trial design. The primary goals of this study were to examine whether different cognitive trajectories can be identified within subjects with MCI and, if present, to characterize each trajectory in relation to changes in all major Alzheimer’s disease (AD) biomarkers over time.

**Methods:**

Individuals with a diagnosis of MCI at the first visit and ≥ 1 follow-up cognitive assessment were selected from the Alzheimer’s Disease Neuroimaging Initiative database (*n* = 936; age 73 ± 8; 40% female; 16 ± 3 years of education; 50% APOE4 carriers). Based on the Alzheimer’s Disease Assessment Scale-Cognitive Subscale-13 (ADAS-Cog-13) total scores from baseline up to 5 years follow-up, a non-parametric *k*-means longitudinal clustering method was performed to obtain clusters of individuals with similar patterns of cognitive decline. We further conducted a series of linear mixed-effects models to study the associations of cluster membership with longitudinal changes in other cognitive measures, neurodegeneration, and in vivo AD pathologies.

**Results:**

Four distinct cognitive trajectories emerged. Cluster 1 consisted of 255 individuals (27%) with a nearly non-existent rate of change in the ADAS-Cog-13 over 5 years of follow-up and a healthy-looking biomarker profile. Individuals in the cluster 2 (*n* = 336, 35%) and 3 (*n* = 240, 26%) groups showed relatively mild and moderate cognitive decline trajectories, respectively. Cluster 4, comprising about 11% of our study sample (*n* = 105), exhibited an aggressive cognitive decline trajectory and was characterized by a pronouncedly abnormal biomarker profile.

**Conclusions:**

Individuals with MCI show substantial heterogeneity in cognitive decline. Our findings may potentially contribute to improved trial design and patient stratification.

**Supplementary Information:**

The online version contains supplementary material available at10.1186/s13195-023-01205-w.

## Background

Mild cognitive impairment (MCI) is often thought of as a transitional stage between normal cognitive aging and dementia, including Alzheimer’s disease (AD) [1, 2]. However, MCI is linked to substantial biological heterogeneity [3, 4]. Although the annual rate of conversion from MCI to AD is set at approximately 10–12% [5–7], not all who have a diagnosis of MCI demonstrate progressive decline, and many exhibit differential clinical outcomes, including remaining at the MCI level or reverting to cognitively normal (CN) state [8, 9]. In spite of its ubiquity, the heterogeneity of MCI regarding cognitive trajectories and progression to AD is currently not well understood, hindering further progress in clinical practice and research.

To date, there are no effective therapies for the disease, and most clinical trials, aimed to slow down or halt the conversion from MCI to dementia, have so far failed [10]. One possible reason for widespread failures of therapeutic development for the disease may be due to neglecting the heterogeneous nature of MCI and treating all individuals as if they were the same. In line with this notion, a recent simulation study has shown that individuals with early AD demonstrated substantially varying rates of cognitive decline, even when randomization procedures were applied at the start of the study [11]. For instance, one potential way to facilitate the development of effective therapies is to identify and remove subgroups of MCI individuals who have relatively normal cognitive performance and a low rate of progression to AD [12]. The authors suggest that the inclusion of subjects with “disease-free” MCI may attenuate the potential beneficial effects of treatment.

Several previous studies have utilized subtyping approaches to sort out the heterogeneity of MCI in a non-biased manner [4, 13–19]. With regard to delineating cognitive subtypes, several investigators have utilized data-driven and specifically cluster-analytic approaches based on cross-sectional neuropsychological assessment data. Delano-Wood was one of the first to provide evidence that distinct subgroups of MCI can be empirically derived based on cognitive data using a clinical sample of 70 [20]. Subsequent studies applying empirical methods to neuropsychological test scores have identified multiple MCI subgroups in clinic-based [21–28], community-based [29, 30], and clinical trial [12] samples. However, given the progressive nature of cognitive aging or AD, considering the longitudinal progression and not just a snapshot of the current state may provide a more comprehensive picture of the prodrome stage of the disease. In Xie et al.’s [31] study, group-based trajectory modeling (GBTM) has been performed to identify 5 different longitudinal cognitive trajectories on the Mini-Mental State Examination (MMSE) score in subjects with MCI. In three other studies, by Lee et al. [32], Kim et al. [33], and Kim et al. [34], the GBTM method was applied to discover cognitive trajectories according to Clinical Dementia Rating Sum of Boxes (CDR-SB) in subjects with MCI. The first study by Lee et al. identified two cognitive trajectories (fast decliners and slow decliners), while the other two investigations discovered three cognitive trajectories (stable, slow decliners, and faster decliners). Despite the prominent role of the Alzheimer’s Disease Assessment Scale-Cognitive Subscale 13 (ADAS-Cog-13) in evaluating the efficacy of antidementia medications [35], only one study has attempted to delineate the heterogeneity of cognitive trajectories of ADAS-Cog-13 among 238 participants with amyloid-positive MCI [36]. The identified three clusters showed different cognitive trajectories over time. However, no studies have attempted to uncover MCI heterogeneity based on longitudinal ADAS-Cog-13 scores in both amyloid-positive and amyloid-negative subjects. The inclusion of all MCI subjects regardless of amyloid status is clinically relevant, particularly in a clinical trial targeting other pathobiological pathways other than the amyloid pathway. Additionally, further validation of identified cognitive trajectories using other neuropsychological tests, neurodegeneration, in vivo AD pathologies, and clinical progression may enhance the robustness of the resulting clusters.

Thus, by applying a data-driven, longitudinal clustering analysis approach, we investigated whether distinct cognitive trajectories could be derived within the Alzheimer’s Disease Neuroimaging Initiative (ADNI) MCI cohort and, if present, assessed the associations of trajectory membership with longitudinal changes in all major AD biomarkers.

## Methods

### ADNI database

Data used in the present study were obtained from the ADNI database. The ADNI study was launched in 2003, and its primary goal has been to measure the clinical progression of MCI and early AD by utilizing numerous markers, such as clinical, neuropsychological, and imaging assessments. Recruitment procedures for the ADNI cohort have been described at the following website: www.loni.usc.edu/ADNI, and the ADNI eligibility criteria can be found at the following website: www.adni-info.org/Scientists/ADNIStudyProcedures.html.

### Participants

Our study focuses on the 936 individuals diagnosed with amnestic MCI at baseline and at least one follow-up assessment (with ADAS-Cog-13 administration) in the next 5 years. These follow-up time points were included regardless of the diagnostic status of the subject at these visits. Follow-up visits beyond 5 years after baseline were not included in the analyses. MCI diagnosis was assigned to an individual if he/she met the following criteria: memory complaint, memory impairment as verified by the Logical Memory II subscale (delayed paragraph recall) from the Wechsler Memory Scale-revised (WMS-R), MMSE [37] score of between 24 to 30 (inclusive), CDR [38] score of 0.5, essentially preserved activities of daily living, and absence of AD or dementia. These ADNI MCI criteria are largely consistent with commonly used criteria in clinical trials, such as the Peterson criteria [6].

Each ADNI participant or authorized representative provided written informed consent and the institutional review board of each participating ADNI site approved the ADNI study. This project was also submitted for review to the institutional review board of Wenzhou Seventh People’s Hospital. However, given that this study did not involve contact with human subjects and used de-identified data, the institutional review board of Wenzhou Seventh People’s Hospital determined this study did not require review.

### Neuropsychological tests

ADNI participants underwent a comprehensive battery of neuropsychological assessments during visits. Four primary cognitive tests were selected in this analysis. We included the ADAS-Cog-13 [39], which examines 13 aspects of cognitive function (range: 0–85, higher scores represent more severe cognitive impairment). MMSE, one of the most popular brief screening cognitive tests, was included as a measure of global cognition. The ADNI Memory composite score was derived from MMSE, logical memory, ADAS-Cog (3 versions), and Rey Auditory Verbal Learning Test (2 versions) [40]. The ADNI executive function composite score was developed from Clock Drawing, Digit Span Backwards, Category Fluency, Trails A and B, and Wechsler Adult Intelligence Scale-Revised Digit Symbol Substitution. Both the ADNI Memory and Executive function composite scores have been validated in previously published studies [41, 42].

### Structural magnetic resonance imaging (MRI) measures

The procedure for MRI acquisition has been described previously [43]. Temporal lobe atrophy, particularly in the hippocampal formation and entorhinal cortex, has been considered to be the biological alteration most proximal to the onset of cognitive impairment [44]. Additionally, ventricular enlargement is thought to be a valid measure of clinical progression in individuals with MCI or AD [45]. In this study, we thus focus on these three structural MRI markers. To adjust sex differences in head size, three structural MRI markers were calculated using the following formulas:

$$Adjusted\;hippocampal\;volume\;(aHV)\;=\;hippocampal/intracranial\;volume\;\times\;10^3$$

$$Adjusted\;entorhinal\;cortex\;volume\;(aEV)\;=\;entorhinal\;cortex/intracranial\;volume\;\times\;10^3$$

$$Adjusted\;ventricular\;volume\;(aVV)\;=\;ventricular/intracranial\;volume\;\times\;10^3$$

### PET imaging measures

The cerebral metabolic rate for glucose was determined by [^18^F] fludeoxyglucose (FDG) positron emission tomography (PET). A standard procedure for image pre-processing can be found at the following website: http://adni.loni.usc.edu/methods/pet-analysis/pre-processing/. Previous ADNI studies developed a “MetaROI” of the brain regions that demonstrate hypometabolic alterations among subjects with MCI and AD [46, 47]. These brain regions included bilateral posterior cingulate, right and left angular gyri, and right and left inferior temporal gyri. Global Standardized uptake value ratios (SUVRs) were calculated by averaging FDG uptake across the MetaROI and dividing by the pons and cerebellum.

Brain amyloid deposition was examined by [^18^F] florbetapir (AV45) PET as shown in http://www.adni-info.org. The mean AV45 uptake was determined within four regions, including frontal, lateral parietal, anterior/posterior cingulate, and lateral temporal regions. Global SUVRs were calculated by averaging across four brain regions and dividing by the whole cerebellum.

### Genetic/CSF-based biomarkers

APOE (gene map locus 19Q13.2) genotypes of the study participants were extracted from the ADNI database. Participants with no APOE4 genotype were classified as APOE4 non-carriers while participants with at least one APOE4 genotype were categorized as APOE4 carriers. The levels of CSF Aβ42, total-tau (t-tau), and phosphorylated-tau at threonine 181 (p-tau) were determined by the Department of Pathology & Laboratory Medicine and Center for Neurodegenerative Disease Research, Perelman School of Medicine, University of Pennsylvania. The multiple xMAP Luminex platform and Innogenetics INNO-BIA AlzBio3 immunoassay reagents were used [48].

### Clustering of longitudinal trajectories of cognitive performance

**Fig. 1:**
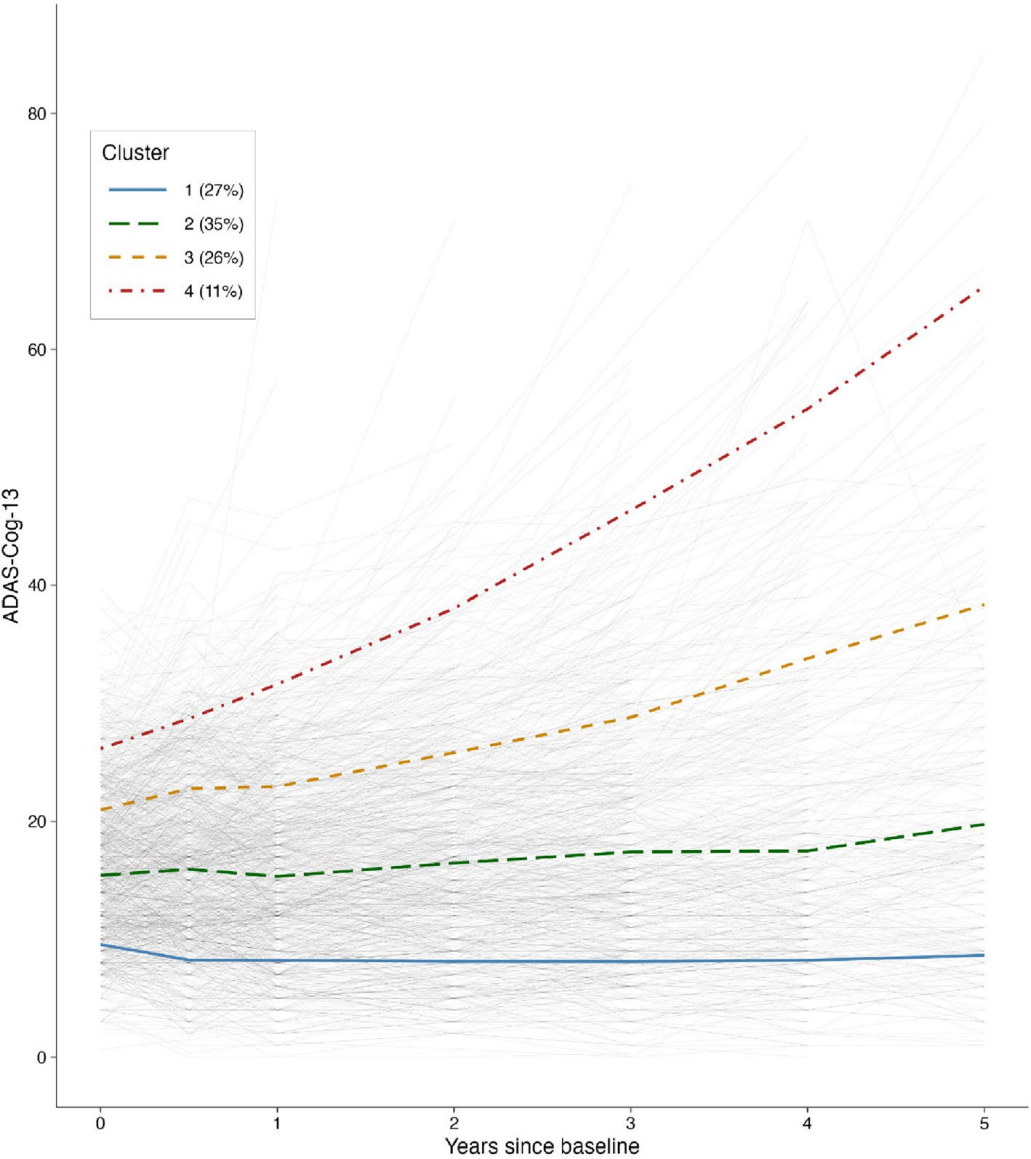
Cognitive trajectories based on ADAS-Cog-13 from baseline to 5 years. Overall, 936 participants with MCI were included in this analysis, including those with at least 2 data points of ADAS-Cog-13 over a 5-year follow-up period. Four cognitive trajectories were identified by the longitudinal K-means cluster analysis. The solid blue, long-dash green, dash orange, and dot-dash red lines represent clusters 1, 2, 3, and 4, respectively. The thin gray lines represent individual cognitive trajectories. Abbreviation: ADAS-Cog-13, Alzheimer’s Disease Assessment Scale-Cognitive Subscale 13

Given that ADAS-Cog is the gold standard for evaluating the efficacy of antidementia medications [49], ADAS-Cog-13 (a modified version of ADAS-Cog that includes more cognitive tasks than the original one and is thought to be more sensitive to cognitive impairment at the earliest stages of dementia) was used as our primary cognitive outcome. Only participants with at least one follow-up assessment of ADAS-Cog-13 were included in this analysis. To identify distinct longitudinal cognitive profiles, a non-parametric *k*-means longitudinal clustering method in the R package “kml” [50] was applied to detect trajectories of cognitive decline over a 5-year follow-up period. Before the clustering analysis, missing values were imputed by the “Copy Mean” method [50]. Briefly, the basic idea of this method is to impute missing values either using linear interpolation or last occurrence carried forward (LOCF) and then add a variation to make the individual trajectory similar to the “shape” of the overall sample’s average trajectory. We built the models for 1 to 8 clusters and selected the 4-cluster solution based on the Bayesian information criterion (BIC) [51] and the elbow method. A visual representation of the elbow method was created (see Additional file 1: Fig. S1). The raw individual trajectories and resultant 4-cluster trajectories are demonstrated in Fig. 1.

### Statistical analyses

**Fig. 2:**
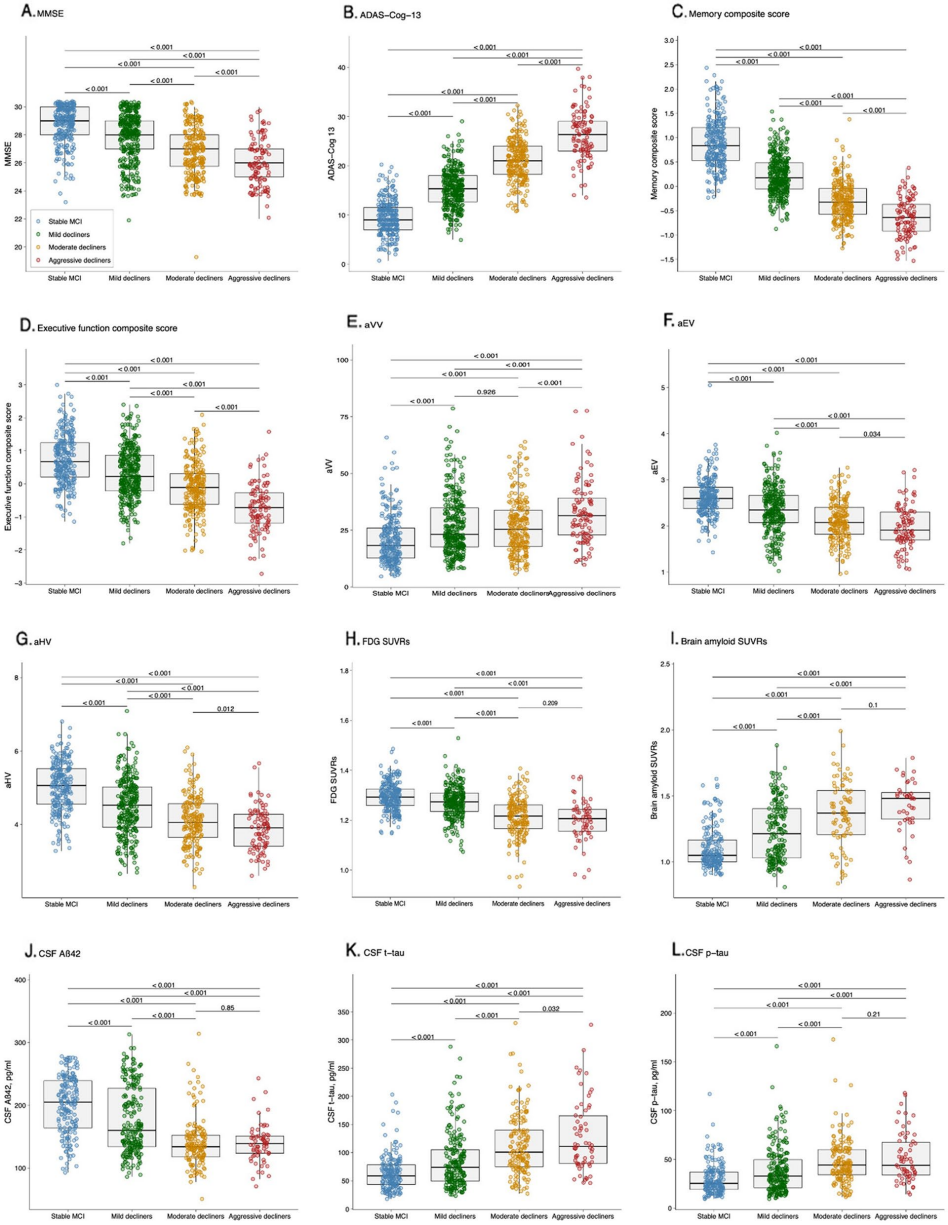
Baseline characteristics by cluster. Differences between clusters were assessed with ANOVA. When group differences were detected with ANOVA, we used pairwise t-tests in post hoc analyses and corrected for multiple testing using the FDR correction. Abbreviations: MCI, mild cognitive impairment; MMSE, Mini-Mental State Examination; ADAS-Cog-13, Alzheimer’s Disease Assessment Scale-Cognitive Subscale 13; aVV, adjusted ventricular volume; aEV, adjusted entorhinal cortex volume; aHV, adjusted hippocampal volume; FDG, fludeoxyglucose; SUVRs, standardized uptake value ratios; Aß42, ß-amyloid; t-tau, total tau; p-tau, phosphorylated tau

At baseline, we used the R statistical software v4.1.2 [52] to explore the relationships between cluster membership and demographics, APOE4 status, neuropsychological evaluations, structural MRI assessments, PET imaging markers, and CSF AD pathologies. The differences between clusters were assessed with analysis of variance (ANOVA) for continuous variables and Pearson’s *x* ^2^ tests for categorical variables. When group differences were detected with ANOVA or Pearson’s *x* ^2^ tests, we used pairwise *t*-tests or *x* ^2^ tests in post hoc analyses and corrected for multiple testing using the false discovery rate (FDR) correction [53]. Group comparisons were also demonstrated visually as shown in Fig. 2.

Linear mixed-effects models were used to examine the associations of cluster membership with the longitudinal change in cognition, structural MRI markers, PET imaging markers, and CSF AD biomarkers over up to 5 years from baseline. Eleven models were created for the following dependent variables: MMSE, memory composite score, executive function composite score, aHV, aEV, aVV, FDG SUVRs, AV45 SUVRs, CSF Aβ42, t-tau, and p-tau. Time since baseline (years), clusters, and their interaction were included as fixed effects. Age, gender, years of education, APOE4 status, and their interactions with time were included as covariates. All models included a random intercept for each participant. The model equations are as follows:

$$Y_{change}\;\sim\;Clusters^\ast time\;+\;Age^\ast time\;+\;Gender^\ast time\;+\;Education^\ast time\;+\;APOE4\;status^\ast time$$

where *Y*_change_ is the change in each dependent variable from the baseline.

Additionally, to further understand the slope differences of these four cluster groups, pairwise comparisons between clusters using the estimated marginal means (EMMs) were performed and the FDR method was used to correct for multiple testing.

Kaplan-Meier curves were conducted to demonstrate the rate of conversion to dementia in the four clusters, and pairwise comparisons using log-rank tests were performed to compare survival curves. Follow-up duration was the number of years from baseline to dementia diagnosis at their last visit. During their follow-up period, subjects who did not convert to dementia were censored at their last visit.

## Results

### Findings of longitudinal k-means cluster analysis

As illustrated in Fig. 1, MCI subjects were assigned into the following clusters according to their cognitive trajectories: (1) cluster 1 with stable cognitive performance (we called this cluster “stable MCI,” *n* = 255, 27%), (2) cluster 2 with a mild cognitive decline (we called it “mild decliners,” *n* = 336, 35%), (3) cluster 3 with a moderate cognitive decline (we called it “moderate decliners,” *n* = 240, 26%), and (4) cluster 4 with a steep cognitive decline (we called it “aggressive decliners,” *n* = 105, 11%).

### Baseline cluster characteristics

Table 1 shows the demographic and clinical characteristics by cluster. For age, the participants in the mild, moderate, and aggressive decliners groups were older than those in the stable MCI group, while no other pairwise difference was found. For education, the participants in the mild, moderate, and aggressive decliners groups were less educated than those in the stable MCI group, while no other pairwise difference was significant. For gender, the mild decliners group had a lower female proportion than the stable MCI group, while no other pairwise difference was observed. For APOE4 status, the participants in the moderate decliners and aggressive decliners groups had a higher proportion of APOE4 carriers than those in the stable MCI and mild decliners groups, while no other pairwise difference was found. For follow-up duration, the stable MCI group had a longer follow-up duration than all other groups, and the aggressive decliners group had a shorter follow-up duration than the mild decliners and moderate decliners groups. Regarding neuropsychological performance, all cognitive tests (MMSE, ADAS-Cog-13, memory composite score, and executive function composite score) showed significant differences between clusters. Regarding structural MRI assessments, four groups showed significant differences, with the exception of equivalent levels between the mild decliners and moderate decliners groups on aVV. For brain glucose metabolism, the moderate decliners and aggressive decliners groups did not differ in FDG SUVRs, while all other pairwise differences were significant. For both brain amyloid PET and CSF Aβ42, all pairwise differences were significant, with the exception of comparable levels between the moderate decliners and aggressive decliners groups. Regarding CSF tau pathologies, all pairwise differences were significant, with the exception of comparable levels between the moderate decliners and aggressive decliners groups on CSF p-tau. Figure 2 showing group differences is also created for visual inspection.

**Table 1:** Cluster characteristics at baseline

Characteristic	Stable MCI (cluster 1), *N* = 255	Mild decliners (cluster 2), *N* = 336	Moderate decliners (cluster 3), *N* = 240	Aggressive decliners (cluster 4), *N* = 105	*p* value
Age, years	70 (7)	74 (8)^a^	74 (7)^a^	74 (7)^a^	< 0.001
Education, years	17 (2)	16 (3)^a^	16 (3)^a^	15 (3)^a^	< 0.001
Gender, *n* (%)					0.020
Male	136 (53%)	221 (66%)	140 (58%)	60 (57%)	
Female	119 (47%)	115 (34%)^a^	100 (42%)	45 (43%)	
APOE4 carriers	93 (36%)	147 (44%)	161 (67%)^a, b^	69 (66%)^a, b^	< 0.001
Follow-up duration, years	3.54 (1.39)	3.29 (1.48)^a^	3.07 (1.40)^a^	2.32 (1.36)^a, b, c^	< 0.001
MMSE	29 (1)	28 (2)^a^	27 (2)^a, b^	26 (2)^a, b, c^	< 0.001
ADAS-Cog-13	10 (3)	15 (4)^a^	21 (4)^a, b^	26 (5)^a, b, c^	< 0.001
Memory composite score	0.88 (0.50)	0.22 (0.40)^a^	− 0.30 (0.40)^a, b^	− 0.64 (0.40)^a, b, c^	< 0.001
Executive function composite score^d^	0.75 (0.76)	0.32 (0.77)^a^	− 0.11 (0.75)^a, b^	− 0.73 (0.73)^a, b, c^	< 0.001
aVV^e^	21 (11)	27 (13)^a^	27 (12)^a^	33 (14)^a, b, c^	< 0.001
aEV^f^	2.62 (0.41)	2.35 (0.51)^a^	2.10 (0.43)^a, b^	1.98 (0.47)^a, b, c^	< 0.001
aHV^g^	5.03 (0.67)	4.48 (0.77)^a^	4.10 (0.68)^a, b^	3.87 (0.60)^a, b, c^	< 0.001
Brain FDG SUVRs^h^	1.29 (0.06)	1.27 (0.06)^a^	1.21 (0.08)^a, b^	1.20 (0.08)^a, b^	< 0.001
Brain amyloid SUVRs^i^	1.10 (0.16)	1.23 (0.22)^a^	1.36 (0.25)^a, b^	1.43 (0.20)^a, b^	< 0.001
CSF Aβ42^j^, pg/ml	200 (46)	176 (54)^a^	140 (39)^a, b^	139 (31)^a, b^	< 0.001
CSF t-tau^k^, pg/ml	64 (31)	85 (49)^a^	114 (55)^a, b^	129 (64)^a, b, c^	< 0.001
CSF p-tau^l^, pg/ml	30 (16)	39 (24)^a^	48 (23)^a, b^	52 (25)^a, b^	< 0.001

*Notes*: Continuous variables are summarized as mean (standard deviation), and categorical variables are summarized as *n* (%). Differences between the clusters were assessed with ANOVA for continuous variables and Pearson’s *x* ^2^ tests for categorical variables. When group differences were detected with ANOVA or Pearson’s *x* ^2^ tests, we used pairwise *t*-tests or *x* ^2^ tests in post hoc analyses and corrected for multiple testing using the FDR correction

*Abbreviations*: *MCI* mild cognitive impairment, *APOE* apolipoprotein E, *MMSE* Mini-Mental State Examination, *ADAS-Cog-13* Alzheimer’s Disease Assessment Scale-Cognitive Subscale 13, *aVV* adjusted ventricular volume, *aEV* adjusted entorhinal cortex volume, *aHV* adjusted hippocampal volume, *FDG* fludeoxyglucose, *SUVRs* standardized uptake value ratios, *Aß42* ß-amyloid, *t-tau* total tau, *p-tau* phosphorylated tau

^a^ *p* < 0.05 compared with stable MCI

^b^ *p* < 0.05 compared with mild decliners

^c^ *p* < 0.05 compared with moderate decliners

^d^Number of participants for executive function analysis: stable MCI: *n* = 255; mild decliners: *n* = 335; moderate decliners: *n* = 240; aggressive decliners: *n* = 104

^e^Number of participants for aVV analysis: stable MCI: *n* = 245; mild decliners: *n* = 321; moderate decliners: *n* = 224; aggressive decliners: *n* = 100

^f^Number of participants for aEV analysis: stable MCI: *n* = 224; mild decliners: *n* = 281; moderate decliners: *n* = 190; aggressive decliners: *n* = 90

^g^Number of participants for aHV analysis: stable MCI: *n* = 228; mild decliners: *n* = 282; moderate decliners: *n* = 191; aggressive decliners: *n* = 89

^h^Number of participants for FDG PET analysis: stable MCI: *n* = 237; mild decliners: *n* = 262; moderate decliners: *n* = 174; aggressive decliners: *n* = 59

^i^Number of participants for AV45 PET analysis: stable MCI: *n* = 182; mild decliners: *n* = 179; moderate decliners: *n* = 83; aggressive decliners: *n* = 41

^j^Number of participants for CSF Aβ42 analysis: stable MCI: *n* = 177; mild decliners: *n* = 213; moderate decliners: *n* = 152; aggressive decliners: *n* = 63

^k^Number of participants for CSF t-tau analysis: stable MCI: *n* = 176; mild decliners: *n* = 208; moderate decliners: *n* = 150; aggressive decliners: *n* = 62

^l^Number of participants for CSF p-tau analysis: stable MCI: *n* = 177; mild decliners: *n* = 213; moderate decliners: *n* = 152; aggressive decliners: *n* = 63

### Cluster membership and longitudinal changes

**Fig. 3:**
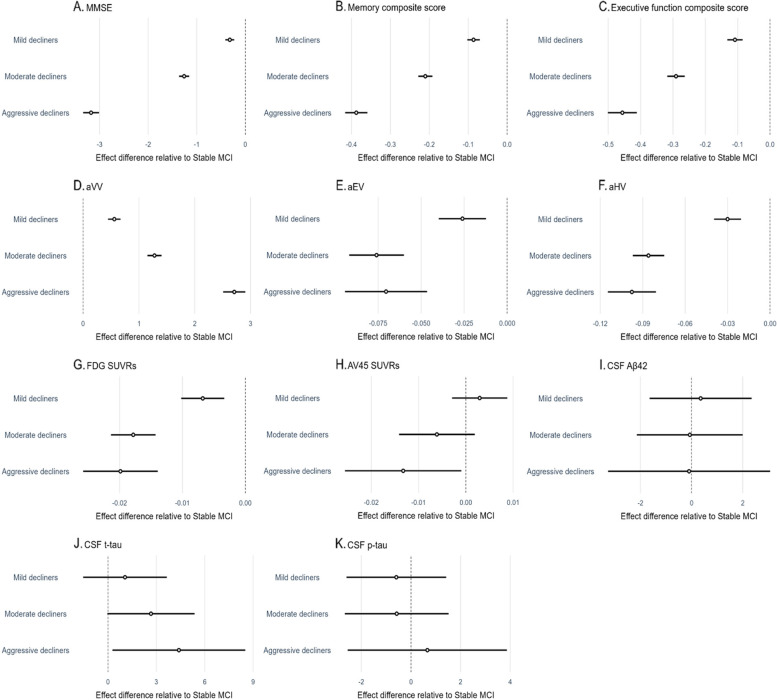
Forest plots showing the effect difference relative to stable MCI. Circles represent coefficients (as shown in Table 2), and horizontal dark lines represent the 95% confidence intervals. Abbreviations: MMSE, Mini-Mental State Examination; aVV, adjusted ventricular volume; aEV, adjusted entorhinal cortex volume; aHV, adjusted hippocampal volume; FDG, fludeoxyglucose; SUVRs, standardized uptake value ratios; Aß42, ß-amyloid; t-tau, total tau; p-tau, phosphorylated tau

**Fig. 4:**
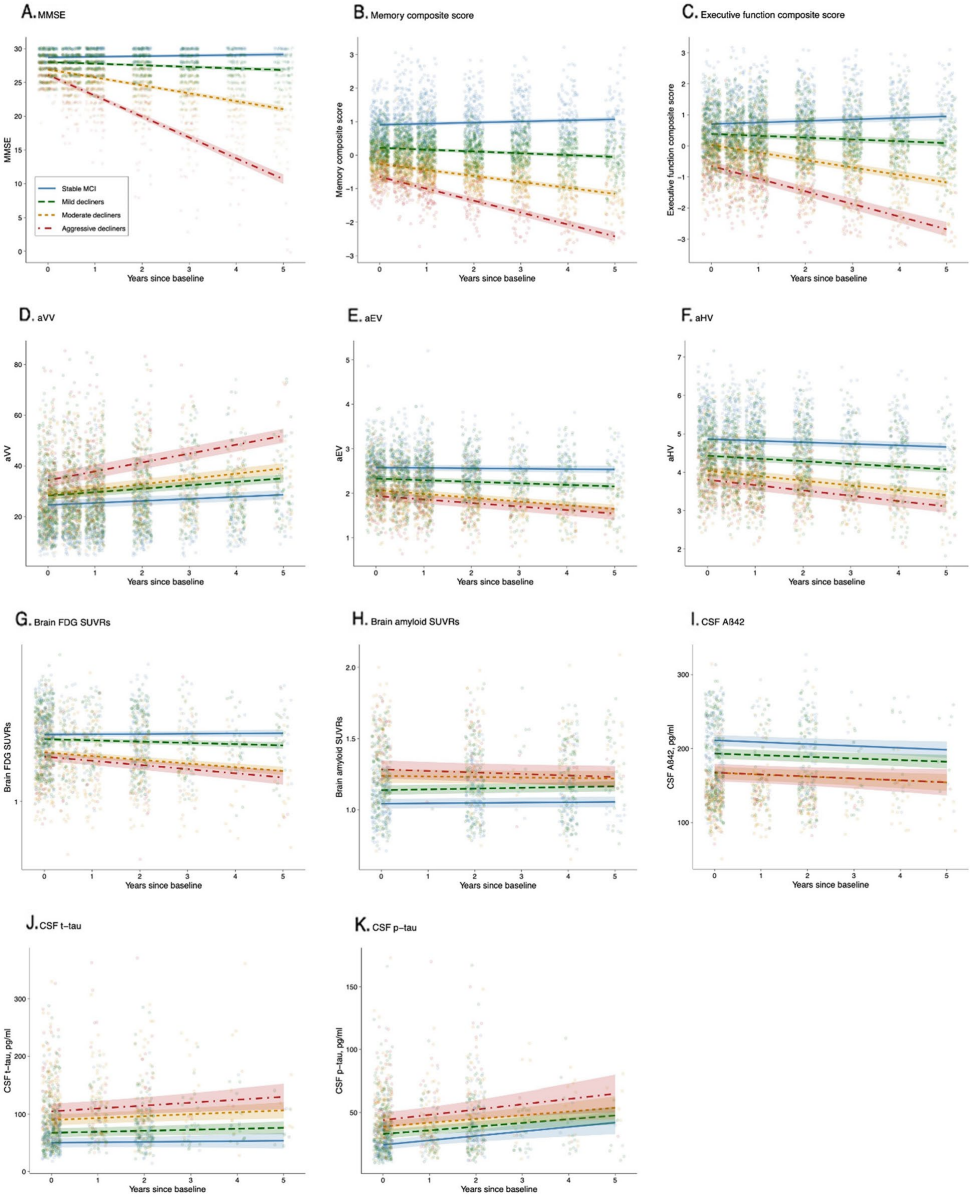
Cluster membership and longitudinal changes in all major AD biomarkers over a 5-year follow-up. Intercepts and slopes of the four clusters come from linear mixed-effects models. There were significant differences between all four clusters in the amount of change in MMSE, memory, executive function, and aVV (A–D). All pairwise differences in the rates of decline in aEV, aHV, and FDG-PET were significant, with the exception of comparable levels between the cluster 3 and 4 groups (E–G). However, four clusters exhibited similar rates of change in Aβ-PET, CSF Aβ42, t-tau, and p-tau proteins (H–K). Abbreviations: MMSE, Mini-Mental State Examination; aVV, adjusted ventricular volume; aEV, adjusted entorhinal cortex volume; aHV, adjusted hippocampal volume; FDG, fludeoxyglucose; SUVRs, standardized uptake value ratios; Aß42, ß-amyloid; t-tau, total tau; p-tau, phosphorylated tau

The results from the linear mixed-effects models examining the associations between cluster membership and longitudinal changes in cognition, neurodegeneration, and CSF AD pathologies are displayed in Table 2 and Figs. 3 and 4.

**Table 2:** Summary of linear mixed-effects models

	**MMSE**			**Memory composite score**			**Executive function composite score**		
**Predictors**	**Coefficients**	**SE**	***p*** **value**	**Coefficients**	**SE**	***p*** **value**	**Coefficients**	**SE**	***p*** **value**
Age × time	0.0011	0.0027	0.6819	-0.0020	0.0005	< 0.0001	-0.0001	0.0007	0.9233
Female gender × time	-0.0025	0.0386	0.5208	-0.0180	0.0067	0.0075	-0.0142	0.0100	0.1554
Education × time	-0.0074	0.0068	0.2791	-0.0029	0.0012	0.0131	-0.0065	0.0018	0.0003
APOE4 carriers × time	-0.1399	0.0389	0.0003	-0.0297	0.0068	< 0.0001	-0.0130	0.0100	0.1959
Mild decliners × time	-0.3193	0.0465	< 0.0001	-0.0865	0.0081	< 0.0001	-0.1084	0.0120	< 0.0001
Moderate decliners × time	-1.2590	0.0536	< 0.0001	-0.2103	0.0093	< 0.0001	-0.2905	0.0138	< 0.0001
Aggressive decliners × time	-3.1750	0.0832	< 0.0001	-0.3877	0.0145	< 0.0001	-0.4563	0.0228	< 0.0001
	**aVV**			**aEV**			**aHV**		
**Predictors**	**Coefficients**	**SE**	***p*** **value**	**Coefficients**	**SE**	***p*** **value**	**Coefficients**	**SE**	***p*** **value**
Age × time	0.0104	0.0033	0.0017	-0.0015	0.0004	0.0006	-0.0013	0.0003	< 0.0001
Female gender × time	-0.0698	0.0476	0.1430	-0.0049	0.0060	0.4102	-0.0228	0.0041	< 0.0001
Education × time	0.0214	0.0082	0.0093	-0.0022	0.0010	0.0319	-0.0007	0.0007	0.3294
APOE4 carriers × time	0.3513	0.0468	< 0.0001	-0.0216	0.0058	0.0002	-0.0248	0.0040	< 0.0001
Mild decliners × time	0.5588	0.0569	< 0.0001	-0.0260	0.0070	0.0002	-0.0301	0.0049	< 0.0001
Moderate decliners × time	1.2790	0.0637	< 0.0001	-0.0759	0.0081	< 0.0001	-0.0859	0.0057	< 0.0001
Aggressive decliners × time	2.7090	0.1011	< 0.0001	-0.0704	0.0122	< 0.0001	-0.0977	0.0086	< 0.0001
	**FDG SUVRs**			**AV45 SUVRs**			**CSF Aβ42**		
**Predictors**	**Coefficients**	**SE**	***p*** **value**	**Coefficients**	**SE**	***p*** **value**	**Coefficients**	**SE**	***p*** **value**
Age × time	0.0001	0.0001	0.3708	-0.0000	0.0002	0.9945	-0.0833	0.0579	0.151
Female gender × time	-0.0019	0.0014	0.1760	0.0013	0.0026	0.6208	-0.2765	0.8175	0.735
Education × time	-0.0003	0.0002	0.1579	0.0009	0.0005	0.0748	-0.0749	0.1469	0.610
APOE4 carriers × time	-0.0030	0.0014	0.0297	0.0104	0.0027	0.0001	1.1217	0.8007	0.162
Mild decliners × time	-0.0068	0.0018	0.0001	0.0029	0.0030	0.3265	0.3555	1.0177	0.727
Moderate decliners × time	-0.0178	0.0018	< 0.0001	-0.0061	0.0041	0.1342	-0.0674	1.0562	0.949
Aggressive decliners × time	-0.0199	0.0030	< 0.0001	-0.0133	0.0063	0.0351	-0.0975	1.6161	0.952
	**CSF t-tau**			**CSF p-tau**				\	
**Predictors**	**Coefficients**	**SE**	***p*** **value**	**Coefficients**	**SE**	***p*** **value**	\	\	\
Age × time	-0.0441	0.0754	0.5593	-0.0794	0.0584	0.1746	\	\	\
Female gender × time	1.0084	1.0639	0.3437	-0.2223	0.8223	0.7870	\	\	\
Education × time	0.4866	0.1919	0.0115	0.0218	0.1480	0.8831	\	\	\
APOE4 carriers × time	-1.0515	1.0448	0.3147	-0.7189	0.8105	0.3755	\	\	\
Mild decliners × time	1.0575	1.3234	0.4246	-0.5903	1.0249	0.5649	\	\	\
Moderate decliners × time	2.6670	1.3778	0.0535	-0.5745	1.0674	0.5906	\	\	\
Aggressive decliners × time	4.3979	2.1002	0.0367	0.6619	1.6368	0.6861	\	\	\

*Notes*: The main effects of predictors (i.e., age, gender, years of education, APOE4 status, cluster membership, and years since baseline) are included in all models, while coefficients are not shown for the sake of brevity. Coefficients are unstandardized values, which represent the magnitude of change in each AD biomarker yearly

*Abbreviations*: *MMSE* Mini-Mental State Examination, *aVV* adjusted ventricular volume, *aEV* adjusted entorhinal cortex volume, *aHV* adjusted hippocampal volume, *FDG* fludeoxyglucose, *SUVRs* standardized uptake value ratios, *AV45* florbetapir, *Aß42* ß-amyloid, *t-tau* total tau, *p-tau* phosphorylated tau

For the models involving cognitive assessments (MMSE, the ADNI memory composite score, and the ADNI executive function composite score; see Table 2, Figs. 3A–C and 4A–C), the clusters (mild decliners, moderate decliners, and aggressive decliners) × time interactions were all significant such that the mild decliners, moderate decliners, and aggressive decliners groups had steeper slopes (i.e., faster cognitive decline) compared to the stable MCI group. To further understand the group differences in slope, post hoc analyses using the FDR correction were performed. The group differences in slope were significant in all pairwise comparisons (all FDR-adjusted *p* < 0.0001).

For the aVV model (see Table 2, Figs. 3D and 4D), the clusters × time interactions were all significant such that the mild decliners, moderate decliners, and aggressive decliners groups had steeper slopes on aVV (i.e., faster ventricular enlargement) compared to the stable MCI group. To further understand the group differences in slope, post hoc analyses using the FDR correction were performed. The group differences in slope were significant in all pairwise comparisons (all FDR-adjusted *p* < 0.0001).

For the aEV and aHV models (see Table 2, Figs. 3E, F and 4E, F), the clusters × time interactions were all significant such that the mild decliners, moderate decliners, and aggressive decliners groups had steeper slopes (i.e., faster entorhinal atrophy and faster hippocampal atrophy) compared to the stable MCI group. To further understand the group differences in slope, post hoc analyses using the FDR correction were performed. All pairwise differences in slope were significant, with the exception of comparable levels between the moderate decliners and aggressive decliners groups on entorhinal atrophy (coefficient: − 0.00551, SE: 0.01223, FDR-adjusted *p*: 0.6526) and on hippocampal atrophy (coefficient: 0.0117, SE: 0.0087, FDR-adjusted *p*: 0.1798).

For the FDG SUVRs model (see Table 2, Figs. 3G and 4G), the clusters × time interactions were all significant such that the mild decliners, moderate decliners, and aggressive decliners groups had steeper slopes on FDG SUVRs (i.e., faster decline in brain glucose metabolism) compared to the stable MCI group. To further understand the group differences in slope, post hoc analyses using the FDR correction were performed. All pairwise differences in slope were significant, with the exception of comparable levels between the moderate decliners and aggressive decliners groups on FDG SUVRs (coefficient: 0.0020, SE: 0.0030, FDR-adjusted *p*: 0.4963).

For the AV45 SUVRs model (see Table 2, Figs. 3H and 4H), the aggressive decliners × time term, but not the mild decliners × time or moderate decliners × time term, was significant, indicating that the aggressive decliners group rather than the mild decliners or moderate decliners group differed in slopes on AV45 SUVRs compared to the stable MCI group. When corrected by the FDR method, however, post hoc analyses did not find any pairwise differences in slopes (all FDR-adjusted *p* > 0.05).

For the CSF Aß42 model (see Table 2, Figs. 3I and 4I), the clusters × time interactions were not significant, suggesting that the mild decliners, moderate decliners, and aggressive decliners groups did not differ in slopes on CSF Aß42 compared to the stable MCI group. Furthermore, post hoc analyses also did not find any pairwise differences in slopes (all FDR-adjusted *p* > 0.05).

For the CSF t-tau model (see Table 2, Figs. 3J and 4J), the aggressive decliners × time term, but not the mild decliners × time or moderate decliners × time term, was significant, indicating that the aggressive decliners group rather than the mild decliners or moderate decliners group had a steeper slope on CSF t-tau compared to the stable MCI group. When corrected by the FDR method, however, post hoc analyses did not find any pairwise differences in slopes (all FDR-adjusted *p* > 0.05).

For the CSF p-tau model (see Table 2, Figs. 3K and 4K), the clusters × time interactions were not significant, suggesting that the mild decliners, moderate decliners, and aggressive decliners groups did not differ in slopes on CSF p-tau compared to the stable MCI group. Furthermore, post hoc analyses also did not find any pairwise differences in slopes (all FDR-adjusted *p* > 0.05).

### Progression to dementia

**Fig. 5:**
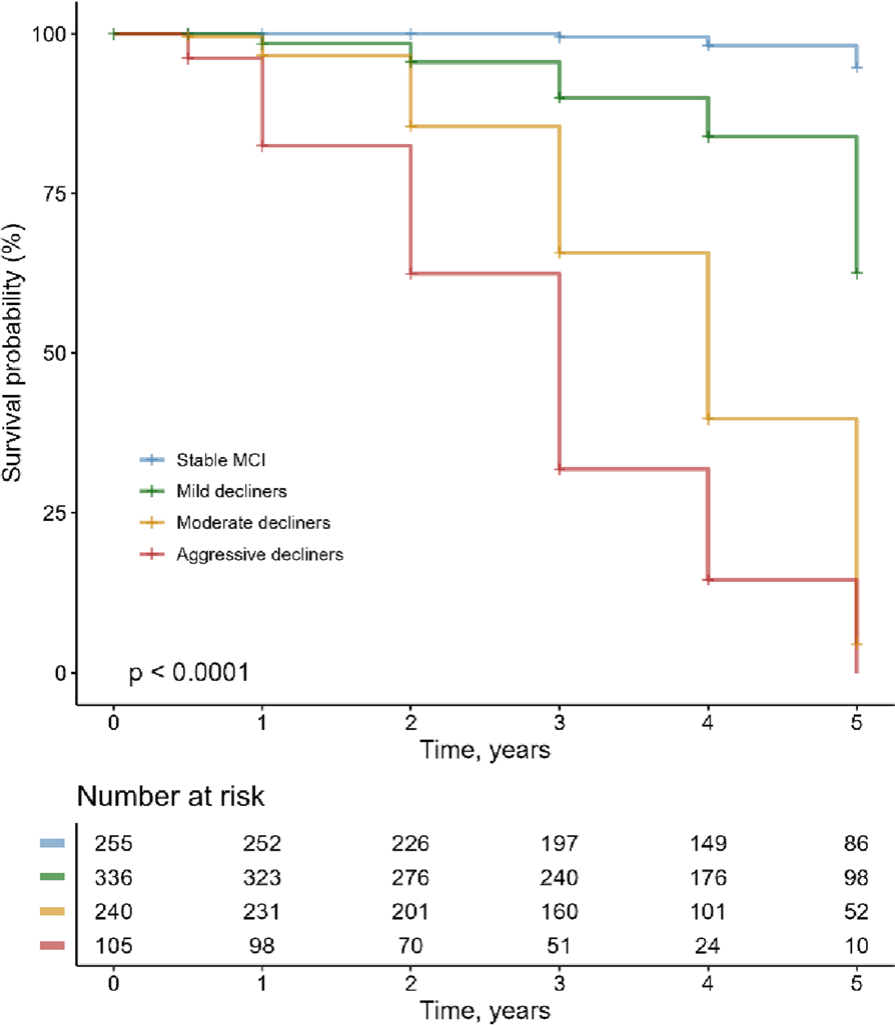
Kaplan-Meier survival curves showing the rate of progression to dementia in the four clusters. All clusters differed significantly from one another. Abbreviation: MCI, mild cognitive impairment

Of the 936 subjects, 307 (32.8%) progressed to dementia within a 5-year follow-up period. Kaplan-Meier curves showing the rate of conversion to dementia are demonstrated in Fig. 5. A log-rank test found significant cluster differences in survival curves (*x* ^2^[3] = 462; *p* < 0.001). All pairwise comparisons with the FDR correction were significant (FDR-adjusted *p* < 0.001). Regarding the type of dementia, 294 of the 307 (95.8%) who converted to dementia were diagnosed with AD dementia. Thirteen subjects (4.2%) progressed to a non-AD dementia (4 frontal temporal dementia, 3 primary progressive aphasia, 1 progressive supranuclear palsy, 1 vascular dementia, 1 Shy-Drager syndrome, 1 semantic dementia, 1 with Parkinson’s disease and Lewy body dementia features, 1 other CNS disorder).

### Sensitivity analyses

The residual approach was used to obtain adjusted MRI volumes to examine whether our MRI results are robust to a different ICV adjustment approach [54]. Coefficients and the 95% confidence intervals of the three linear mixed-effects models were summarized (see Additional file 1: Fig. S2). Compared to the results of Fig. 3.D–F, the patterns of coefficients and the 95% confidence intervals of these three models remained unchanged.

## Discussion

There were four key findings of the current study. First, there is substantial heterogeneity in disease progression among MCI subjects despite being intentionally recruited as an independent clinical entity often thought of as the prodromal stage of dementia [6]. Second, we identified a considerable portion of subjects with MCI (cluster 1) showing a very little cognitive decline over 5 years of follow-up. This group of individuals exhibited a remarkably benign-looking biomarker profile. Third, individuals in the cluster 2 and 3 groups demonstrated relatively mild and moderate cognitive decline trajectories, respectively. Fourth, a subgroup of individuals with MCI (cluster 4) exhibited an aggressive cognitive decline trajectory and was characterized by a pronouncedly abnormal biomarker profile.

We found substantial differences between the four clusters in clinical characteristics and longitudinal changes in major AD biomarkers, indicating that our data-driven clustering method categorized MCI subjects into biologically and clinically different subgroups. Cluster 1, comprising about 27% of our study sample, showed a nearly non-existent rate of change in the ADAS-Cog-13 over a 5-year follow-up period. This subgroup had remarkably better baseline performance on the ADAS-Cog-13, MMSE, memory composite, and executive function scores; a more healthy-looking biomarker profile; and a substantially slower change in clinical progression relative to the rest of the MCI group. In agreement with this finding, previously published studies conducted in a population-based sample [30] and the ADNI data set [55] also identified a very similar MCI subgroup, which had less impaired baseline performance on cognitive tasks and a substantially lower risk of clinical progression compared to other MCI groups. This subgroup is especially relevant in clinical trials because the inclusion of “disease-free” MCI subjects may minimize the potential to detect the beneficial effects of treatment for MCI [12]. Edmonds and colleagues suggest that the identification and removal of this subgroup could maximize the capability to observe the treatment effects of new therapeutics in clinical trials involving subjects with MCI [12]. Better stratification of MCI populations before recruitment in clinical trials may help increase the chances of observing efficacy and will contribute to the development of more efficient study designs.

Cluster 2, the largest MCI subgroup identified in this study (*n* = 336, 35%), initially performed worse (indicated by a larger intercept in Fig. 1) on ADAS-Cog-13 and exhibited slightly faster cognitive deterioration over time relative to cluster 1. Moreover, levels of AD-associated biomarkers differed significantly between the cluster 1 and 2 subgroups (Fig. 2). Namely, at baseline, participants in cluster 2 had higher levels of CSF tau pathologies and lower levels of CSF Aβ42 compared to those in cluster 1. These findings are in accordance with earlier longitudinal studies where they have shown that abnormal CSF biomarker profiles are predictive of conversion from MCI to AD with high accuracy [56, 57]. We also found more impaired cognitive performance, as evaluated with the MMSE, memory, and executive function composite scores, in the cluster 2 group, compared to the cluster 1 group (Fig. 2). These two subgroups demonstrated pronounced differences in Aβ-PET SUVRs, a biomarker of amyloid accumulation in the brain, and FDG-PET SUVRs, a marker of neurodegeneration and synaptic dysfunction [58]. Our findings are consistent with a previous PET imaging study, which suggests that Aβ deposition is a robust predictor of clinical progression from MCI to AD [59], with amyloid changes occurring long before the start of cognitive decline [60]. Likewise, the finding that cluster 1 had higher levels of FDG-PET SUVRs (representing less severe neurodegeneration) relative to cluster 2 agrees with the observation that higher levels of FDG-PET SUVRs are associated with remaining cognitively stable among individuals with MCI [61].

Clusters 3 (*n* = 240, 26%) and 4 (*n* = 105, 11%) exhibited substantially steeper cognitive deterioration over time compared to clusters 1 and 2 (Fig. 1), with participants in the cluster 4 group showing the most aggressive cognitive decline trajectory. These findings may have a critical impact on potentiating clinical trials involving MCI subjects based on the predicted magnitude of change in cognition (e.g., ADAS-Cog-13) over time. For example, it is likely that future trials may attempt to enroll those subjects who would be predicted to fall into the cluster 4 group since the inclusion of these aggressive cognitive decliners may enhance the probability of success in clinical trials and lead to a significant gain of power to observe treatment effects. However, this approach should be conducted with caution because cluster 4 is a relatively small group (*n* = 105, 11%). The inclusion of only those subjects in cluster 4 would likely hinder clinical trials, as participants in other subgroups (89%) would be excluded. Furthermore, future studies should focus on the development of statistical models predicting cluster membership using baseline demographics and clinical characteristics to facilitate the recruitment process for clinical trials.

The amyloid cascade hypothesis of AD postulates that the pathologic process initiates with amyloid deposition (as measured by CSF Aβ42 and Aβ-PET), followed by changes in CSF tau proteins, then changes in FDG-PET and structural MRI, followed by cognitive symptoms [44, 62]. Our results largely support this conceptual model. Specifically, our linear mixed-effects models with four cognitive trajectories as the independent variable and all major AD biomarkers as dependent variables (Fig. 4) found that four distinct cognitive decline trajectories (i.e., different rates of cognitive decline) had comparable rates of changes in Aβ-PET, CSF Aβ42, and tau proteins (Fig. 4H–K) but exhibited significantly different rates of changes in structural MRI and FDG-PET (Fig. 4D–G) at the MCI stage of dementia, in accordance with the notion that cognitive decline is only loosely coupled with changes in Aβ-PET and CSF Aβ42 [63, 64], but is tightly accompanied by changes in markers of neurodegeneration [65–67] at the later stages of the disease (e.g., MCI and AD dementia). For instance, previous studies found that among patients who were experiencing a rapid cognitive decline (e.g., AD patients), rates of MRI changes were correlated with cognitive deterioration, while rates of brain amyloid deposition were not [64, 68]. In addition, Vemuri and colleagues found that among subjects with MCI, correlations with cognitive measures were strong with MRI volumes but were not significant with levels of CSF tau [69]. As expected, participants with four distinct cognitive decline trajectories identified by the cluster technique based on longitudinal ADAS-Cog-13 data also exhibited different rates of other cognitive outcomes (i.e., MMSE, memory, and executive function; Fig. 4A–C), and all pairwise differences in rates of cognitive decline were significant. Somewhat unexpectedly, we observed a significant difference between cluster 3 and cluster 4 in the rate of change in aVV (i.e., widening ventricles; Fig. 4D), but not aEV, aHV, or FDG-PET (Fig. 4E–G), despite that all of these imaging markers are thought of as neurodegenerative markers. This discrepancy may be attributed to the fact that relative to medial temporal atrophy (i.e., aEV and aHV) or hypometabolism on FDG-PET, the enlargement of ventricles is considered to be a more downstream event and more strongly coupled with a change in global cognition over time [70]. It is also likely that several factors that we did not examine in the present study, such as cognitive reserve, brain resilience, and other brain neuropathologies (e.g., vascular damages, Lewy bodies), may contribute to the difference in the rate of cognitive decline between clusters 3 and 4 [71, 72].

This study has several limitations. First, we observed highly variable individual trajectories on the ADAS-Cog-13 among individuals with MCI (i.e., the thin gray lines in Fig. 1). In this study, the usage of cluster analysis should be interpreted as an exploratory analysis in nature, rather than a confirmative one. We acknowledge that a larger sample size of each subgroup, particularly the cluster 4 group, would be warranted to yield more robust and generalizable findings. However, our linear mixed-effects models with cluster membership as the independent variable and other cognitive outcomes (i.e., MMSE, memory, and executive function) produced a very consistent pattern of cognitive trajectories (Fig. 4A–C), further supporting the notion that the four trajectories identified in the cluster analysis were stable and robust. Second, we did not use or incorporate other AD biological biomarkers in the clustering process, since our primary study goal was to examine the heterogeneity in cognitive decline, and the ADAS-Cog-13 is the most predominant assessment used to track disease progression in AD clinical trials [49]. Third, changes in AD biomarkers over long periods are non-linear [62] but were modeled as linear in our linear mixed-effects models. Nevertheless, over a shorter period, changes in AD biomarkers can be modeled as linear functions since such non-linearity seems to be minimal [73]. Fourth, the ADNI memory composite score was derived from several memory assessments, such as memory tasks of ADAS-Cog. This may introduce some degree of circularity since the ADNI memory composite score partly overlaps with the ADAS-Cog-13.

In conclusion, we identified four distinct cognitive decline trajectories of MCI and further characterized changes in all major AD biomarkers over time for each subgroup. Our findings highlight the importance of considering the heterogeneity of MCI when recruiting participants in clinical trials, thus potentially contributing to better trial design and more precise personalized medicine.

## Supplementary Information

**Additional file 1.**

## Data Availability

Data used in the present study has been made publicly available by the ADNI in the Laboratory of Neuro Imaging (LONI) database.
