# Supplementary material for: Uncovering heterogeneous cognitive trajectories in mild cognitive impairment: a data-driven approach
Source: Alzheimers Res Ther. 2023 Mar 20;15:57. doi: 10.1186/s13195-023-01205-w (PMC10026406; doi:10.1186/s13195-023-01205-w)
Supplement: Supplementary file 1 — Additional file 1. [file 13195_2023_1205_MOESM1_ESM.docx]

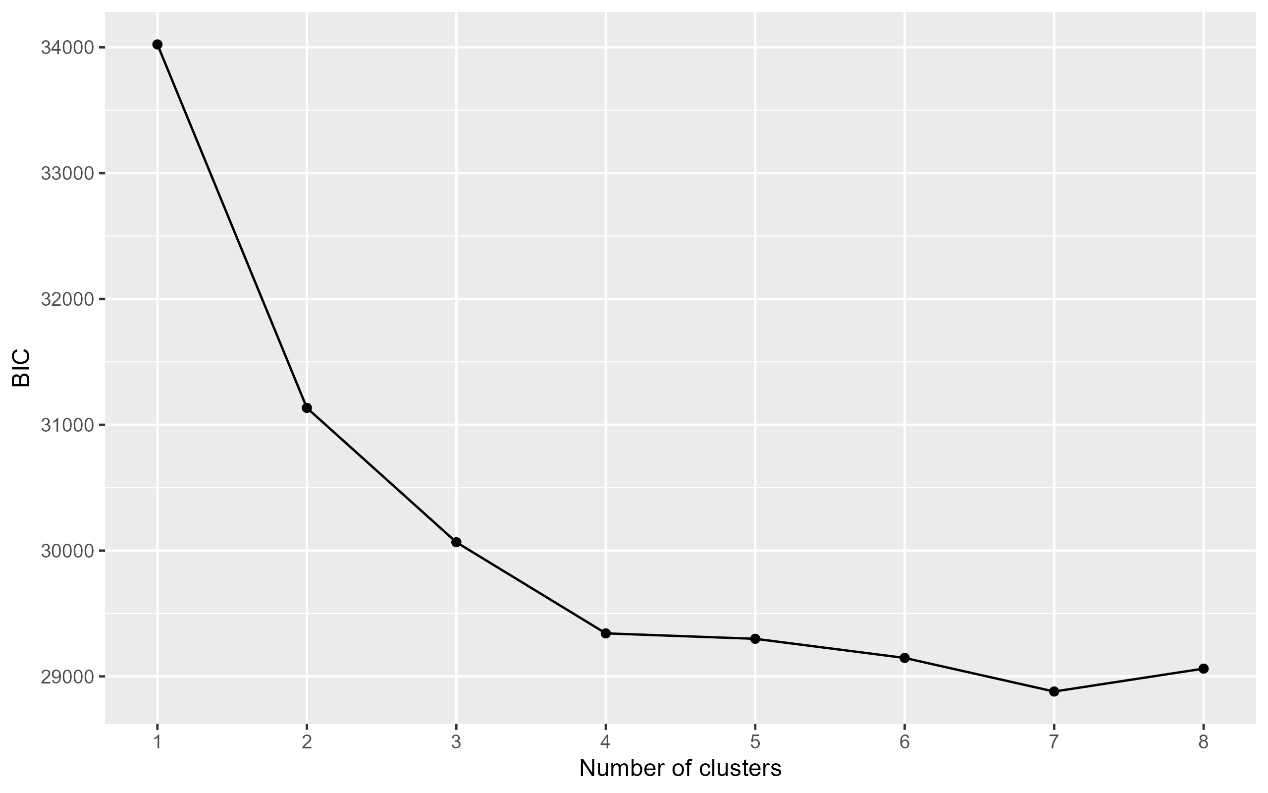


**Supplementary Figure 1**. A visual representation of the elbow method. The inflection point can be identified when the number of clusters is 4.


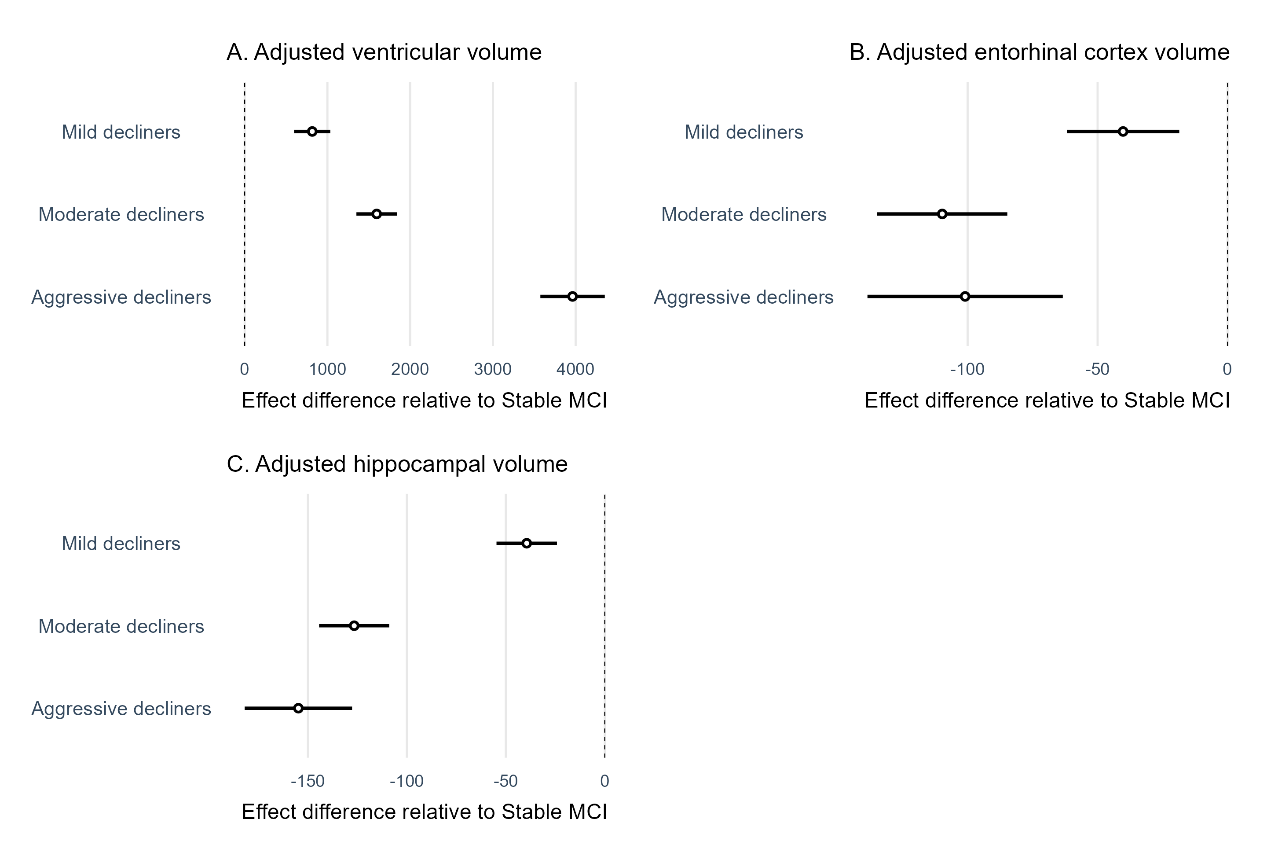


**Supplementary Figure 2**. Forest plots showing effect difference relative to Stable MCI.
